# Acute Diarrheal Disease Associated With Climate Variability and Water Supply System Configuration: An Ecological Study in Brazil

**DOI:** 10.1002/wer.70563

**Published:** 2026-09-09

**Authors:** Isabel Francisco de Araújo Reis, Paula Cristine Silva Gomes, Thaís Barreto Santana, Lívia Cristina Pinto Dias, Marcelo de Souza Lauretto, Maria Tereza Pepe Razzolini, Aníbal da Fonseca Santiago

**Affiliations:** ^1^ Graduate Program in Environmental Engineering Federal University of Ouro Preto Ouro Preto Minas Gerais Brazil; ^2^ Department of Environmental Engineering Federal University of Ouro Preto Ouro Preto Minas Gerais Brazil; ^3^ Department of School of Arts, Sciences and Humanities University of São Paulo São Paulo São Paulo Brazil; ^4^ Department of Environmental Health, School of Public Health University of São Paulo São Paulo São Paulo Brazil; ^5^ Department of Civil Engineering Federal University of Ouro Preto Ouro Preto Minas Gerais Brazil

**Keywords:** diarrhea, low‐ and middle‐income countries, risk assessment, safe water, water safety plan

## Abstract

Acute diarrheal disease (ADD) remains a major public health problem in low‐ and middle‐income countries, where vulnerabilities in water supply systems (WSSs) are associated with increased exposure to disease. Rainfall patterns influenced by climate change are also associated with changes in ADD occurrence, although evidence on the interaction between ADD, WSS configurations, and climate variability remains limited. This ecological study used water quality indicators, health surveillance records, and generalized linear models to assess associations between different WSS configurations, including conventional treatment systems, disinfection systems, and systems distributing a mixture of treated and raw water. The exponentiated coefficient for systems distributing a mixture of treated and raw water was exp(*β*) = 1.21 (95% CI: 1.03–1.43; *p* = 0.032), indicating that ADD rates were 21% higher in these systems than in conventional treatment systems. Similarly, the exponentiated coefficient for the dry season was exp(*β*) = 0.73 (95% CI: 0.62–0.86; *p* = 0.001), indicating that ADD rates were 27% lower during the dry season than during the rainy season. Water quality indicators showed no statistically significant association with ADD cases. These findings highlight the importance of improving water treatment infrastructure, particularly in hybrid WSSs, to reduce ADD rates and protect public health.

## Graphical abstract

Acute diarrheal disease rates are associated with seasonality and the type of water supply system.

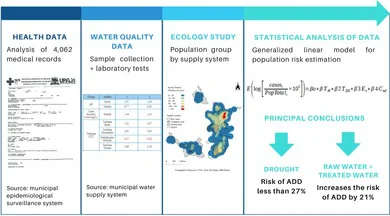

## Summary

- Understanding water treatment systems can guide preventive public health actions.
- The risk of acute diarrheal diseases (ADDs) was 27% lower during the dry season than during the rainy season.
- Mixing raw and treated water was associated with a 21% higher ADD rate compared with conventional water treatment systems.
- Water supply system configuration was associated with differences in ADD rates.
- Hybrid water supply systems were associated with higher ADD rates than conventional treatment systems.

## 1 Introduction

Despite advances in mitigation strategies, acute diarrheal diseases (ADDs) remain a significant global public health problem, especially in low‐ and middle‐income countries, where they ranked among the leading causes of mortality in 2021 (World Health Organization [WHO] 2024). These infections are indicative of environmental conditions related to water quality (Vieira 2018) and are associated with structural limitations such as inadequate infrastructure, intermittent water supply, and failures in water treatment and distribution processes (World Health Organization 2014).

Intermittent water supply systems (WSSs) are associated with several operational challenges, including pressure fluctuations, negative‐pressure events, contaminant intrusion through leaks, deterioration of water quality, and difficulties in maintaining adequate disinfectant residuals throughout the distribution network (Kumpel and Nelson 2016). WSSs that rely on disinfection as the sole treatment process, without conventional treatment steps such as coagulation, flocculation, sedimentation, and filtration, may have limitations in the inactivation of some microorganisms, making appropriate operational control essential (Orner et al. 2017).

However, the health impacts associated with these different water supply configurations remain underexplored. Intermittent systems and those relying solely on disinfection are more common in low‐ and middle‐income countries, reflecting varying levels of water service, ranging from the provision of adequately treated water to the use of untreated sources, especially in regions with greater socioeconomic vulnerability (United Nations Children's Fund and World Health Organization 2023).

In urban areas outside major metropolitan centers, the health risks associated with drinking water may not be fully captured. As noted by Santos et al. (2023), part of the population may be supplied through irregular connections, which are associated with an increased risk of contamination. This situation may be exacerbated by the presence of alternative water supply solutions and by lower coverage of water treatment services in certain localities (Brasil. Ministério da Saúde. Fundação Nacional de Saúde 2019).

Research on water‐related risks has largely focused on conventional WSS, as demonstrated by Sato et al. (2013) and Bataiero et al. (2019). Thus, the assessment of health risks associated with different WSS configurations in low‐ and middle‐income countries represents a significant gap in the literature. Although the association between unsafe water and ADD is well established (Ercumen et al. 2014; Prüss‐Ustün et al. 2014), important gaps remain in understanding how different supply configurations influence these conditions, as well as the role of seasonal variations in this process. Particularly noteworthy are systems based solely on disinfection (without filtration) or those supplied by a combination of treated and raw water.

In addition, precipitation and surface runoff have been linked to outbreaks of ADD, a relationship investigated by Curriero et al. (2001). Environmental and climatic changes may further influence the transmission dynamics of infectious diseases by modifying environmental conditions, pathogen persistence, and human exposure, thereby increasing the burden of climate‐sensitive diseases (Uwishema et al. 2023). Evidence from a study conducted in Malawi indicates that extreme weather events can damage water, sanitation, and hygiene (WASH) infrastructure, increase contamination of water sources, and contribute to outbreaks of waterborne diseases (Focus et al. 2023). These impacts may be exacerbated in the context of climate change. According to Su et al. (2025), the global average temperature in 2023 surpassed the previous record set in 2016, reaching 1.45°C ± 0.12°C above the 1850–1900 average and approaching the 1.5°C limit established by the Paris Agreement relative to pre‐industrial levels. In 2024, this threshold was temporarily exceeded. At the current rate, there is an estimated 50% probability that global warming will exceed 1.5°C within approximately 6 years. This warming has been associated with increases in the frequency and intensity of natural disasters (Su et al. 2025). Given this scenario, prioritizing adaptation strategies is essential, particularly in light of existing gaps in the implementation of measures aimed at adapting water supply solutions.

Climate change may be associated with an increase in the frequency of precipitation events; therefore, this study sought to quantify the relationship between climate seasonality and the number of cases of ADD in the real‐world context of a middle‐income country, where limitations in sanitation and water supply services may exacerbate impacts on public health. We also investigated how different configurations of WSS and water quality at the point of consumption influence the risk of ADD, considering the context of structural and operational vulnerabilities.

Specifically, the study compared the risks among populations exposed to water supplied by systems based on disinfection, distribution networks with a mixed supply of treated and raw water, and systems using conventional treatment. By analyzing these configurations under real‐world conditions, the study contributes to a better understanding of the risks associated with heterogeneous WSS and provides insights for the implementation of preventive measures in vulnerable contexts, particularly in the face of climate change.

## 2 Materials and Methods

All stages of this study adhered to ethical research principles and were approved by the Research Ethics Committee of the Federal University of Ouro Preto (UFOP), under the Certificate of Presentation for Ethical Consideration (CAAE) protocol number 80122524.7000.5150.

### 2.1 Study Area

For this study, the municipality of Mariana, located in the state of Minas Gerais, Brazil, was selected because it represents a context in which different WSS coexist, reflecting structural inequalities in the provision of water treatment services (SAAE 2025). Understanding the challenges faced by this municipality allows for comparisons with those identified in other countries with similar contexts.

Water collection, treatment, and distribution services in Mariana are managed by the Autonomous Water and Sewerage Service (SAAE), a municipal agency. The study area corresponds to the urban zone of the municipality of Mariana, encompassing the main district and the district of Passagem de Mariana, with an estimated population of 49,204 inhabitants based on data from the 2022 Brazilian Census (Brazilian Institute of Geography and Statistics [IBGE] 2023). Among this population, 4062 residents were diagnosed with ADD in 2023, according to the Secretaria Municipal de Saúde (2023), resulting in a rate of 8255 cases of ADD per 100,000 inhabitants.

**FIGURE 1:**
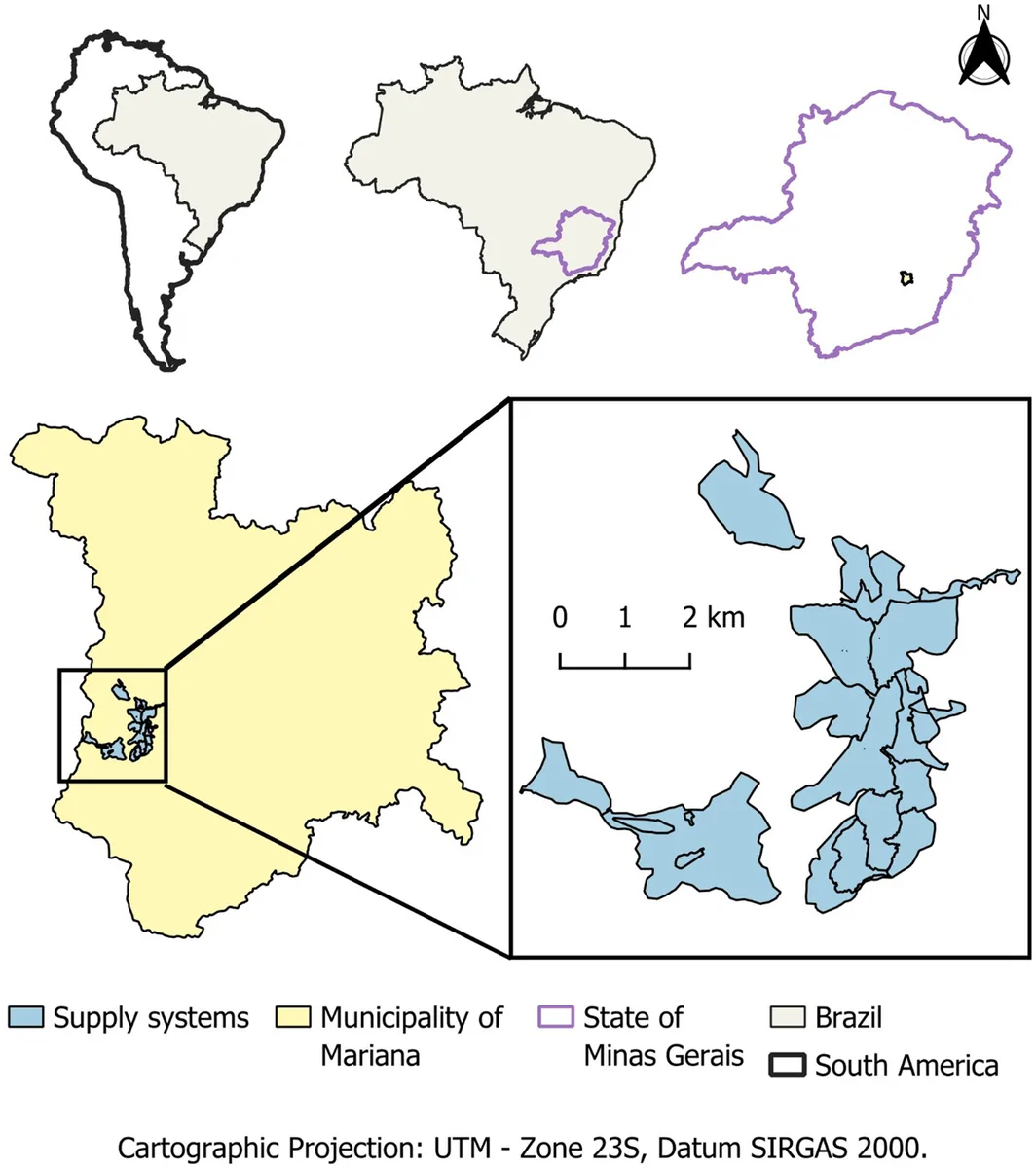
Location of the municipality of Mariana.

Figure 1 shows the location of the municipality of Mariana, Minas Gerais, and the distribution of WSS in the study area.

With regard to the public water supply, approximately 220 L/s are currently abstracted from surface water and groundwater sources. Surface water sources include intake points located in well‐preserved areas of the Atlantic Forest within the Gualaxo do Sul River and Ribeirão do Carmo watersheds (Souza 2004).

Water is supplied to the population through 17 WSSs. Treatment is provided either by conventional treatment systems, which include coagulation, flocculation, sedimentation, filtration, and disinfection, or by disinfection‐only systems based exclusively on chlorination, without the preceding stages of conventional treatment. However, in systems with structural limitations, intermittent operation, or limited operational control capacity, treated water may mix with raw water within the distribution network. In this study, these systems were classified as hybrid WSSs. In addition to these systems, the population living in areas without formal land tenure receives untreated (raw) water.

Table 1 summarizes the main characteristics of the WSSs in Mariana and the population served. In hybrid WSSs, where water is treated exclusively by disinfection, chlorination is performed, on average, twice a week by a single operator responsible for multiple systems. This limitation may hinder timely adjustment of chlorine dosage and the maintenance of adequate free residual chlorine levels, potentially increasing microbiological vulnerability, particularly during the rainy season.

**TABLE 1:** Characteristics of the WSS.

WSS	Classification	System description	Water source	Number of samples^a^	Population^b^
SCC ETA Sul, SCC ETA Santa Rita de Cássia e SCC ETA Matadouro	Conventional	Water is treated through the stages of coagulation, flocculation, sedimentation, filtration, and disinfection.	Surface	13	11,188
SDS Efigênia; SDS Pantera; SDS Liberdade, SDS Cartuxa Dulico, SDS Maquiné Inconfidentes, SDS Gogo	Disinfection	Water is treated exclusively with chlorine; no filtration is performed.	Surface/groundwater	30	16,804
SH ETA Sul, SH ETA Matadouro; SH ETA Seminário, SH ETA Santa Rita, SH Del Rey, SH Cristal Maquiné	Hybrid	Part of the water is treated by conventional treatment or simple disinfection. These systems allow, within the distribution network, the mixing of raw water, water treated by simple disinfection, and water treated by conventional treatment.	Surface/groundwater	49	20,427
Cristais Raw Water WSS Serrinha Raw Water WSS	Untreated	Raw water distributed without any treatment.	Surface/groundwater	0	785

^a^ Number of samples collected monthly in the distribution network (pH, apparent color, turbidity, total coliforms, and *E. coli* ) during the monitoring period.

^b^ Estimated population supplied by each WSS.

### 2.2 Ecological Study Design

This study adopted a population‐based ecological design to evaluate the association between ADD occurrence, seasonality, and the characteristics of WSSs in the urban area of Mariana, Minas Gerais, Brazil. Secondary data were obtained from the Municipal Health Department of Mariana and included all reported ADD cases recorded between January and December 2023 among residents of the study area, totaling 4062 records. Each record contained the patient's residential address, allowing georeferencing and assignment of each case to its corresponding WSS, resulting in a raw database comprising 4062 individual records.

To estimate the population served by each WSS, spatial data from the Brazilian Institute of Geography and Statistics (IBGE) census tracts, municipal neighborhood boundaries, and WSS service areas were integrated using QGIS version 3.34.9. Because the boundaries of the WSS did not coincide with census tract boundaries, the population served by each system was estimated using an area‐weighted proportional allocation method. Specifically, the population of each census tract was redistributed according to the proportion of its area located within each WSS, allowing estimation of the population potentially served by conventional WSS, disinfection‐only WSSs, hybrid WSSs, and raw water WSS.

Based on the date of disease occurrence, individual records were aggregated by WSS and classified according to seasonality (rainy or dry season). The ecological units of analysis consisted of combinations of WSS and seasonality, resulting in 30 observations (15 WSS × 2 seasonal periods). For each observation, the following variables were obtained: WSS, WSS type (disinfection‐only, conventional, and hybrid), number of ADD cases, seasonality, population served, the ADD rate per 100,000 inhabitants, and the base‐10 logarithm of this rate (LogT), which was used as the response variable in the statistical modeling. This dataset was designated as the analytical dataset and is provided in Supporting Information S1.

Water quality information was incorporated into the analytical dataset using the Frequency of Out‐of‐Specification Analyses (*iAF*) indicator, calculated for each WSS and seasonal period as the number of noncompliant analyses per 1000 analyses performed, considering free residual chlorine, turbidity, and *Escherichia coli* (Equation 1). The *iAF* was included as an explanatory variable together with WSS type and seasonality.

Because the analysis was conducted using data aggregated by WSS and seasonality rather than daily case counts, no observations with zero ADD cases were present in the analytical dataset. Observations corresponding to raw water WSSs were excluded from the statistical modeling because no water quality monitoring data were available for these systems, making it impossible to calculate this indicator.

### 2.3 Health Data

**FIGURE 2:**
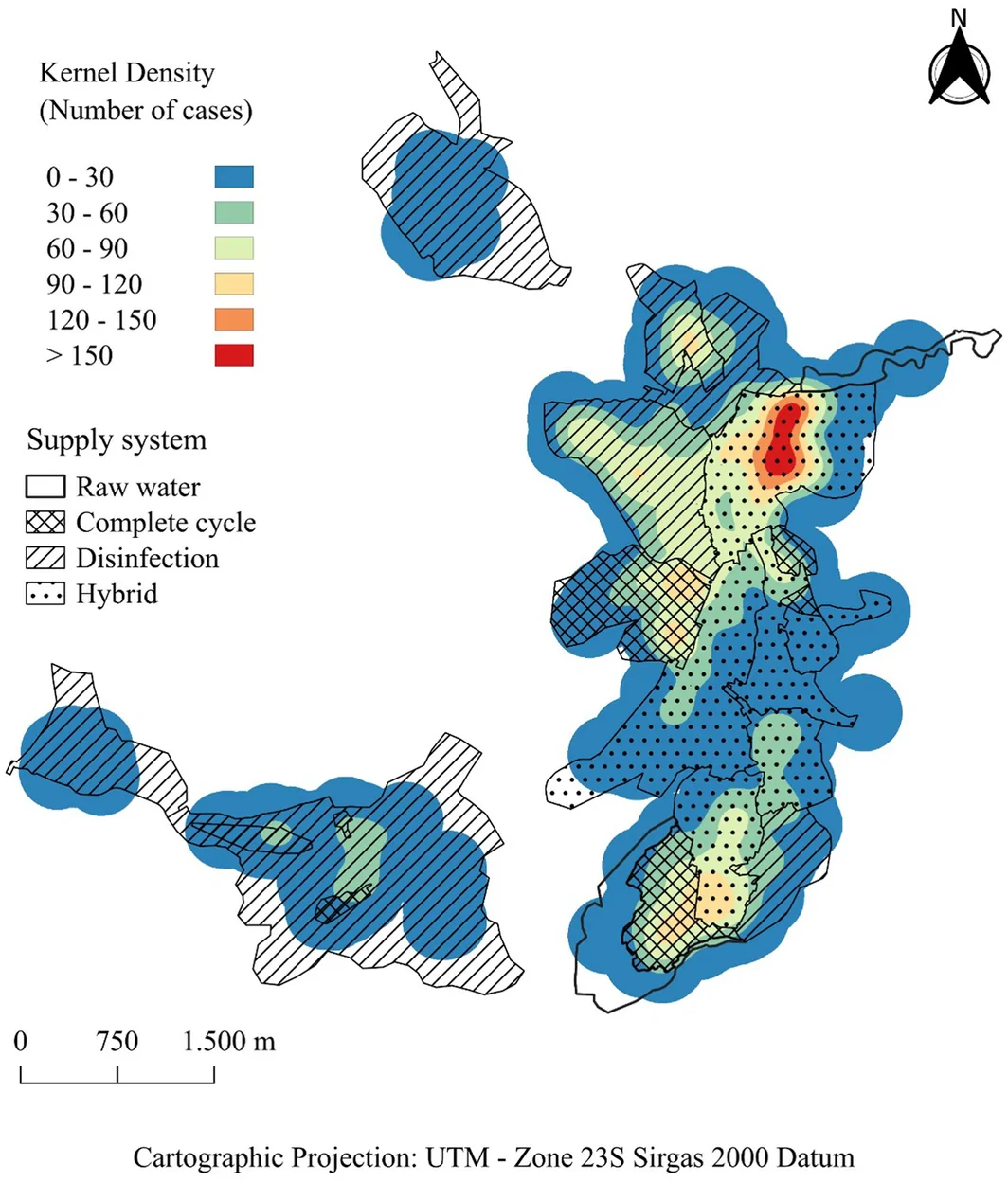
Spatial distribution of ADD cases occurring in Mariana, Minas Gerais, in 2023.

Cases of ADD were defined by the Municipal Health Department based on the presence of at least one of the following symptoms: watery diarrhea, vomiting, nausea, or abdominal pain. The addresses were geocoded and linked to WSS. Spatial analysis was performed in QGIS version 3.34.9, using a Kernel density estimate (200‐m radius), ensuring data anonymity. Figure 2 shows the populations within their respective WSS.

### 2.4 Water Quality

After defining the WSS, the results of analyses for turbidity, free residual chlorine, and *E. coli* conducted during the dry season (April to September) and the rainy season (October to March) of 2023 were collected. Only water quality data from the distribution network were used, since the evaluated parameters were common to all assessed system configurations.

Table 1 presents the number of sampling points for water intended for human consumption and the sampling frequency. The locations of the sampling points were determined by the water supply service provider, which is responsible for monitoring, based on the sampling plan established according to the recommendations of the Brazilian Ministry of Health, taking into account the population served by each WSS.

Data from 92 sampling points distributed across the evaluated systems were used (Table 1). Based on monthly collections conducted over a 12‐month period in 2023, a total of 1104 samples were initially expected. However, the number of collections exceeded this estimate, reaching 2301 samples, since additional sampling is required whenever results do not comply with potability standards. These additional analyses include new collections at the contaminated sampling point and at adjacent points in the distribution network, located upstream and downstream, as established by Ordinance GM/MS No. 888/2021 (Brasil 2021).

The World Health Organization (WHO) recommends operational parameters to ensure the microbiological safety of water, including maintaining a minimum free residual chlorine concentration of 0.2 mg/L in the distribution system, the absence of *E. coli* , and turbidity ≤ 1 NTU after filtration, thereby ensuring effective disinfection (WHO 2022b).

In Brazil, Ministry of Health (MS) Ordinance No. 888/2021 establishes potability standards for water intended for human consumption, including the absence of *E. coli* , a minimum free residual chlorine concentration of 0.2 mg/L in the distribution network, and turbidity limits that vary according to the stage of the WSS. For filtered water, turbidity ≤ 0.5 NTU is required in 95% of samples, whereas in the distribution network, values up to 5.0 NTU may be accepted as an operational and organoleptic control parameter. In this study, nonconformities were defined as turbidity values exceeding 5.0 NTU, the presence of *E. coli* , and free residual chlorine concentrations below 0.2 mg/L or above 5.0 mg/L.

The frequency of noncompliance was calculated as the number of noncompliant analyses per 1000 water quality analyses performed, separately for the dry and rainy seasons, according to Equation (1).

(1) $$\mathrm{iAF}=\frac{\text{TAFP}}{\mathrm{TAR}}\times 1.000,$$

where

*iAF*: frequency of noncompliant analyses.

TAFP = total of noncompliant analyses (sum of *E. coli* tests out of standard (iecol) + turbidity tests out of standard (it) + free residual chlorine tests out of standard (ic)).

TAR = total number of analyses performed (sum of iecol + it + ic conducted).

Correlation tests were performed between the *iAF* indicator and the number of ADD cases per 100,000 inhabitants, using Spearman's correlation coefficient, given the nonnormal distribution of the variables. The analyses were conducted on the entire dataset and were stratified by dry and rainy seasons, using a significance level of 5%.

### 2.5 Statistical Analysis

Initially, Poisson and negative binomial regression models were fitted using the number of ADD cases as the response variable and the logarithm of the population served as an offset. The explanatory variables included the frequency of noncompliant analyses (*IAF*), water supply system type, and seasonality. Detailed information on the count regression models and their diagnostic evaluation is presented in Supporting Information S2.

Model comparison indicated better performance of the negative binomial model; however, residual diagnostics revealed significant underdispersion. Therefore, a generalized linear model (GLM) with a Gaussian distribution and identity link function was evaluated.

In the GLM, the response variable was defined as the base‐10 logarithm of the ADD case rate per 100,000 inhabitants (LogT). Different model specifications were evaluated, considering the individual and joint contributions of the explanatory variables. Model selection was based on the Akaike Information Criterion (AIC), Bayesian Information Criterion (BIC), deviance, coefficient of determination (*R* ^2^), and statistical significance of the coefficients, also considering the study hypothesis and epidemiological plausibility. The results of the different model specifications and their respective fit criteria are presented in Supporting Information S3. The final model included the hybrid water supply system and dry season.

The adequacy of the final model was assessed through residual diagnostics, which indicated that the model assumptions were adequately satisfied. Statistical significance was defined as *p* < 0.05, and the significance of the coefficients was assessed using the Wald test. Because the response variable was log_10_‐transformed, the regression coefficients were back‐transformed to allow interpretation of changes in the ADD case rate on the original scale.

The final model is presented in Equation (2):

(2) $$\log\left(10^{5}\right)=\beta_{0}+\beta_{1}T_{H}+\beta_{2}E_{c}+\varepsilon_{i}$$

where

$\text{loglog}\;\left(10^{5}\right)$: base‐10 logarithm of the ADD case rate per 100,000 inhabitants in group *i* (dependent variable);

*β*_0_: model intercept;

$\beta_{1},\beta_{2}$: coefficients associated with the independent variables;

$T_{H}$: indicator variable for hybrid systems;

$E_{c}$: indicator variable for the dry season;

$\varepsilon_{i}$: random error term, assumed to follow a normal distribution with mean zero and constant variance.

### 2.6 Declaration on Generative AI and AI‐Assisted Technologies in the Manuscript Preparation Process

During the preparation of this work, the authors used ChatGPT (OpenAI) to assist with the translation of the manuscript from Portuguese to English. After using this tool, the authors reviewed and edited the content as necessary and take full responsibility for the content of the published article.

## 3 Results

Figure 2 shows the spatial distribution of waterborne disease cases in the municipality of Mariana, Minas Gerais, in 2023, showing a higher concentration of cases in the northern part of the municipality, where hybrid systems predominate. A second area of high case density was observed in regions supplied by conventional systems.

**FIGURE 3:**
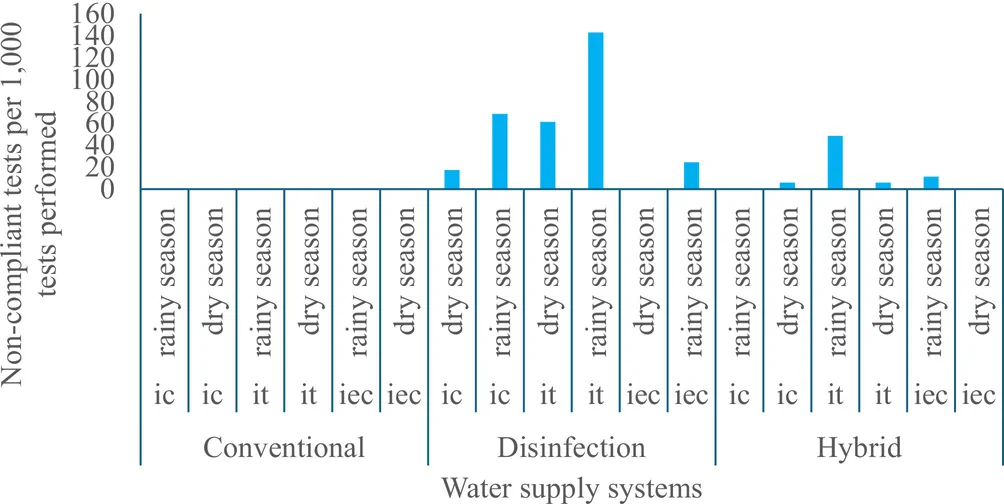
Frequency of turbidity, free residual chlorine, and *Escherichia coli* tests exceeding regulatory standards per 1000 tests performed in 2023. ic = free residual chlorine tests exceeding regulatory standards; iecol = *Escherichia coli* concentration exceeding regulatory standards; it = turbidity tests exceeding regulatory standards.

Figure 3 presents the results of noncompliance tests for turbidity, free residual chlorine, and *E. coli* , conducted during the dry and rainy seasons in 2023. Hybrid systems showed the highest number of noncompliances, followed by simple disinfection systems, whereas conventional systems did not record any such occurrences. Turbidity and *E. coli* exhibited higher frequencies of noncompliance during the rainy season in both hybrid and simple disinfection systems. Free residual chlorine, in turn, showed noncompliance in hybrid systems during the dry season and in simple disinfection systems during the rainy season.

Table 2 presents the results of the final GLM with a Gaussian (normal) distribution and an identity link function, using the log‐transformed ADD rate per 100,000 inhabitants as the response variable. The final model included seasonality (dry season) and the hybrid WSS as independent variables. During model selection, the *iAF* and the disinfection system variables were not statistically significant and were therefore excluded from the final model. The exponentiated coefficients [exp(*β*)] represent multiplicative effects on ADD rates after back‐transformation from the logarithmic scale.

**TABLE 2:** Results of the final generalized linear model (Gaussian distribution with identity link), including seasonality (dry season) and WSS type as explanatory variables for log‐transformed ADD rates.

Variable	Coefficient (*β*)	Standard error	*t* value	*p*	exp(*β*)	95% CI lower	95% CI upper
Intercept	3.610	0.070	53.450	< 0.001	36.820	32.260	42.020
Hybrid	0.190	0.080	2.260	0.032	1.210	1.030	1.430
Dry season	−0.310	0.080	−3.710	0.001	0.730	0.620	0.860

*Note:* Only the final model is presented. The results for the *iAF* and simple disinfection system variables, excluded from the final model due to a lack of statistical significance, are available in Supporting Information S1.

In the GLM with a Gaussian (normal) distribution and an identity link function, the exponentiated coefficient for systems distributing a mixture of treated and raw water was exp(*β*) = 1.21 (95% CI: 1.03–1.43; *p* = 0.032), indicating that ADD rates were 21% higher in these systems than in conventional treatment systems. Similarly, the exponentiated coefficient for the dry season was exp(*β*) = 0.73 (95% CI: 0.62–0.86; *p* = 0.001), indicating that ADD rates were 27% lower during the dry season than during the rainy season.

On the other hand, the disinfection system and *iAF* did not show a statistically significant association with the outcome, providing no evidence of an association between water quality indicators measured in the distribution network and ADD occurrence. Additionally, the correlation analysis indicated a positive and moderate association during the rainy season (*ρ* = 0.44; *p* = 0.10; *n* = 30) and a very weak negative association during the dry season (*ρ* = −0.044; *p* = 0.875; *n* = 30), both without statistical significance.

**FIGURE 4:**
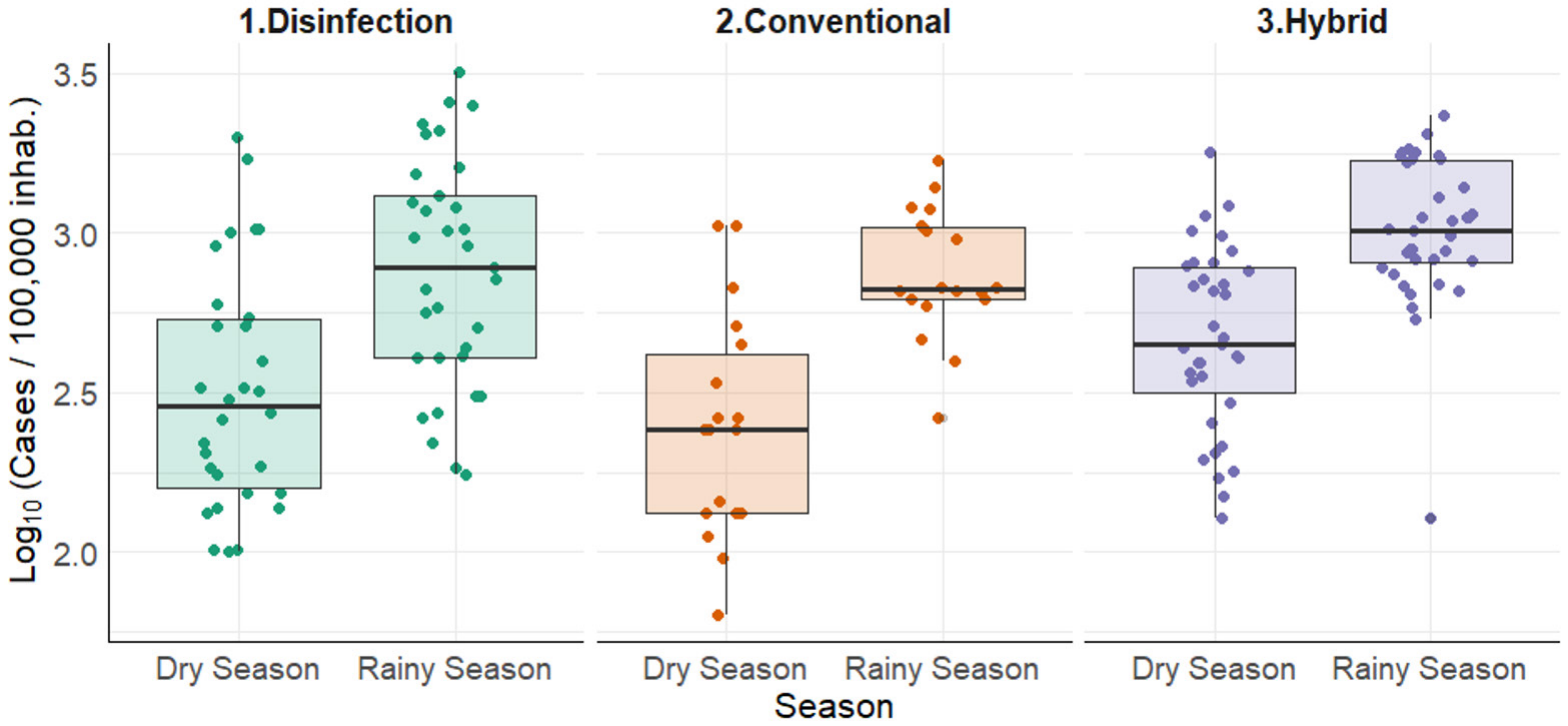
Distribution and variability of ADD cases per 100,000 inhabitants in 2023 in the municipality of Mariana, according to the seasonal period and types of WSS.

Figure 4 shows a higher ADD rate during the rainy season across all evaluated systems. Hybrid systems presented the highest rates, followed by disinfection systems, while conventional systems recorded the lowest rates. Conventional systems showed lower rates compared to hybrid and disinfection systems, with increases in ADD rates observed during the rainy season.

**FIGURE 5:**
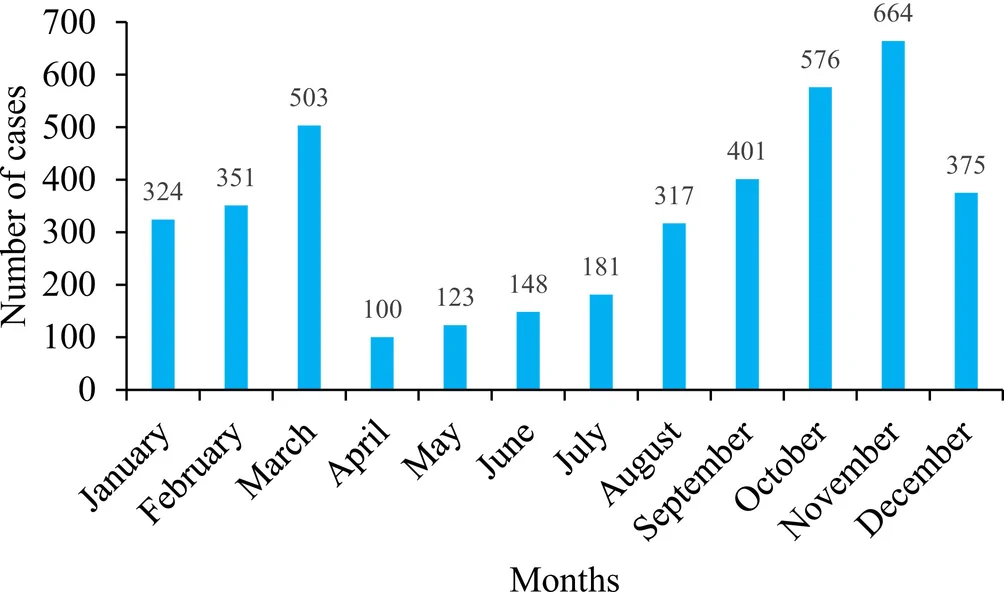
Monthly distribution of total cases of ADD among SUS patients in 2023.

Figure 5 presents the monthly distribution of ADD cases in 2023, showing an increase between October and November and between February and March, coinciding with the beginning and end of the rainy season. This pattern was observed during periods of seasonal variation in precipitation, especially considering the increase recorded in March 2023 (Centro Nacional de Monitoramento e Alertas de Desastres Naturais 2025). In contrast, a reduction in cases is observed between December and February compared to peak periods.

## 4 Discussion

### 4.1 Drinking Water Quality

The fitted model and its parameters presented in Table 2 indicated no statistically significant association between water quality in the distribution network (*iAF*) and the ADD rate. This result is consistent with Vieira (2018), who highlights that biological indicators and compliance‐based approaches may not fully capture contamination dynamics in drinking water systems. This finding supports the relevance of a water safety management approach in WSS.

The absence of a statistically significant association between water quality and ADD was also observed in the correlation analyses of this study, which showed low‐ to moderate‐magnitude associations that were not statistically significant. Similarly, studies conducted in WSS in Pouso Redondo, a municipality in southern Brazil (Santa Catarina State), and in the Federal District, where Brazil's capital is located, also failed to identify a statistically significant association between water quality parameters, including turbidity, free residual chlorine, total coliforms, and noncompliant *E. coli* levels, and the occurrence of diarrheal diseases based on correlation analyses (de Castro et al. 2019; Meisen et al. 2011).

However, the absence of statistical significance should be interpreted with caution, as it may be more related to limitations of the indicator used than to the absence of an association between the variables. Since the *iAF* is based on noncompliance records, it emphasizes instances where parameters exceed established limits, adopting a dichotomous approach (compliance/noncompliance), which reduces variability and may limit sensitivity to detect associations with health outcomes.

A study conducted in Vitória, Espírito Santo, identified statistically significant associations between water quality parameters and the occurrence of ADD using correlation analyses, including strong correlations for turbidity and free residual chlorine and moderate correlations for total and thermotolerant coliforms (Queiroz et al. 2009). This finding may be related to the use of continuous water quality variables, which allow for the incorporation of measurement variability and increase the sensitivity of the analysis to detect statistically significant associations.

It was also observed that conventional systems performed better in maintaining water quality, with no noncompliant results for *E. coli* , turbidity, or free residual chlorine at either station, as shown in Figure 4. This finding is consistent with the literature, which reports greater efficiency of conventional systems in removing contaminants (Santos et al. 2023). In disinfection and hybrid systems, a seasonal pattern was observed, with an increase in noncompliance during the rainy season for turbidity and *E. coli* . This pattern is consistent with increased surface runoff and higher contaminant loads during rainfall periods (Al Mamoon et al. 2019). As an exception, free residual chlorine in hybrid systems showed a higher frequency of noncompliance during the dry season. This result indicates that noncompliance may not be associated solely with seasonality but also with operational factors such as disinfection failures, intermittent supply, or recontamination within the distribution network (Kumpel and Nelson 2016). Thus, monitoring water quality parameters can support corrective decision‐making and contribute to interventions aimed at ensuring water safety management.

### 4.2 Water Supply Systems and the Risk of ADD

In Mariana, 4062 ADD cases were reported through the health surveillance system in 2023, corresponding to an annual ADD notification rate of 8255 cases per 100,000 inhabitants. This notification rate was higher than estimates from the Global Burden of Disease (GBD) study (IHME 2021) for high‐income countries, such as the United States (2740 cases per 100,000 inhabitants) and Canada (5200 cases per 100,000 inhabitants). In contrast, the rate of ADD observed in Mariana was lower than estimates for middle‐income countries, such as Bolivia (19,010 cases per 100,000 inhabitants) and Indonesia (57,450 cases per 100,000 inhabitants). It was also lower than estimates for low‐income countries, such as Chad (167,430 cases per 100,000 inhabitants), Niger (125,150 cases per 100,000 inhabitants), and Somalia (88,730 cases per 100,000 inhabitants).

Although these comparisons help contextualize the magnitude of ADD cases in Mariana relative to different international scenarios, direct comparisons between surveillance‐based notification rates and GBD estimates should be interpreted with caution. The present study is based on cases reported through the local health surveillance system. In contrast, GBD estimates are derived from epidemiological modeling approaches that integrate multiple data sources and account for uncertainties related to under‐reporting, under‐diagnosis, and disease burden estimation (GBD 2021 Diarrhoeal Disease Collaborators 2025). Therefore, differences between estimates may reflect not only variations in ADD occurrence but also differences in surveillance coverage, healthcare access, diagnostic practices, case ascertainment, and methodological approaches.

In this context, the occurrence of ADD may be related to characteristics of WSS that influence patterns of population exposure (Ashbolt 2015). The highest estimated ADD rates were associated with populations served by hybrid systems in the adjusted model presented in Table 2, compared with those served by conventional systems. This finding may be linked to operational configurations in conventional systems that reduce the likelihood of mixing between raw and treated water. Since the raw and treated water distribution networks in conventional systems are physically separated over long distances, interconnections between these networks become less likely, thereby reducing potential exposure to contamination events.

Additionally, although the municipality aims to maintain continuous water supply, some systems may experience intermittent operation due to planned rotational distribution practices or unplanned operational interruptions, such as pipeline failures and maintenance activities. Intermittent supply conditions may increase the vulnerability of distribution networks to water quality deterioration through pressure fluctuations and potential intrusion of contaminants; however, this factor was not directly evaluated in the present study (Kumpel and Nelson 2016).

However, even in areas served by conventional systems, an increase in ADD cases was observed during the rainy season. This trend may be associated with changes in raw water quality during rainfall periods, particularly due to increased turbidity, which increases the load of suspended particles and places greater demand on coagulation, flocculation, and filtration processes.

According to Santos et al. (2021), under conditions of hydraulic overload, inadequate adjustments in coagulant dosing may compromise treatment efficiency, allowing particles to pass through and reducing filtration performance, which may result in lower removal efficiency under high turbidity conditions. In this context, the importance of standardizing operational procedures at treatment plants is emphasized, as this can support appropriate responses under such conditions.

Another factor contributing to the higher estimated rates observed in hybrid systems, compared with conventional systems, is their greater susceptibility to operational failures. This condition is often related to supply interruptions, involving breaks in water supply during the integration of raw and treated water networks, followed by the restoration of flow accompanied by pressure fluctuations. According to LeChevallier et al. (2003), such variations are consistent with potential intrusion of contaminants into distribution systems.

In addition, hybrid systems may experience operational failures in controlling free residual chlorine and disruptions in the distribution network, which may facilitate conditions favorable to the entry of microorganisms. These conditions, as described by Egorov et al. (2002) and Tinker et al. (2009), have been reported to increase microbiological vulnerability by reducing free residual chlorine along the distribution network and, consequently, may be associated with higher observed ADD rates in populations served by these systems.

In a study conducted in Malawi, vulnerabilities in water supply and sanitation infrastructure during an extreme climate event were associated with increased contamination of water sources, contributing to outbreaks of waterborne diseases (Focus et al. 2023). In the present study, hybrid WSS exhibited higher rates of ADD, indicating greater vulnerability compared with conventional WSS.

These findings reinforce the need for investments to improve the infrastructure and operational performance of hybrid WSS in Mariana. This interpretation is consistent with the findings of Ferreira et al. (2021), who demonstrated that efficient investments in WSS and sanitation infrastructure substantially reduce hospitalizations due to waterborne diseases, highlighting that strengthening water supply infrastructure is an effective strategy for protecting public health.

Moreover, the implementation of climate‐resilient water safety plans (WSPs), as recommended by the WHO (2022a), may enhance the capacity of water utilities to identify, monitor, and manage risks associated with climate variability. By integrating infrastructure improvements, operational monitoring, source water protection, and preventive risk management, WSPs can increase the resilience of WSS and contribute to reducing the burden of waterborne diseases, particularly in vulnerable systems such as the hybrid WSS evaluated in this study.

Hybrid WSS may be less effective at removing resistant pathogens, such as *Cryptosporidium*, an agent associated with ADD. This is because, in some of these systems, water is treated solely through disinfection, without any filtration steps. According to Betancourt and Rose (2004), chlorine disinfection is not effective in inactivating *Cryptosporidium* oocysts. Studies conducted in Colombia have shown that systems based exclusively on chlorination present infection risks above acceptable levels, reinforcing the importance of integrated treatment processes (Barragán et al. 2022).

In addition to the statistical analysis, the spatial distribution of cases also reveals patterns. The results in Figure 2 indicate higher ADD rates in areas served by hybrid systems. A concentration of cases is observed in the northeastern portion of the study area, classified as a hybrid WSS, a pattern that is also repeated in the southern region, albeit with lower intensity.

Taken together, these results indicate that the vulnerability of hybrid systems may be associated with greater sensitivity to hydrological variability. Although similar patterns are also observed in conventional systems, their greater operational stability may partially mitigate these variations.

In turn, the absence of a statistically significant association between disinfection systems and the ADD rate, presented in the model results in Table 2, may be related to the greater variability of these rates observed in both the dry and rainy seasons, compared to hybrid and conventional systems, as shown in Figure 4. This pattern is characterized by greater data dispersion, with wider interquartile ranges, indicating instability throughout the analyzed period, which may reduce statistical power to detect associations due to increased uncertainty in the estimates. According to Button et al. (2013), increased data variability reduces the precision of estimates, contributing to a decreased ability to identify statistically significant effects.

### 4.3 Seasonality and Contamination Dynamics

Figures 4 and 5 show that seasonality was associated with variation in ADD occurrence, with an increase in cases during the rainy season. This seasonal pattern is consistent with previous evidence showing that climatic factors, particularly temperature variations, are associated with changes in diarrheal disease occurrence (Carlton et al. 2016). These findings suggest that climatic conditions may influence the transmission dynamics of ADD. During the rainy season, increased precipitation may compromise raw water quality through greater surface runoff and the transport of contaminants, thereby increasing population exposure to waterborne pathogens. Consistent with this interpretation, Uwishema et al. (2023) reported that environmental and climatic changes are important drivers of infectious disease transmission because they modify environmental conditions that affect pathogen persistence and human exposure.

Previous systematic reviews have also highlighted that heavy rainfall events and flooding are associated with increased diarrheal disease occurrence, likely due to changes in environmental conditions and increased transport of fecal contaminants into water sources (Levy et al. 2016).

This finding is consistent with the study by Fonseca et al. (2016), which demonstrates a positive relationship between precipitation and diarrheal diseases, as well as with research conducted in the United States (Curriero et al. 2001) and Bangladesh (Ahmed et al. 2005), which linked heavy rainfall to increased transport of fecal contaminants to water sources, potentially increasing the likelihood of exposure to pathogens.

In this context, during the rainy season, increased turbidity may reduce treatment efficiency by shielding microorganisms from the action of chlorine and hindering disinfection (Mazurek et al. 2025). Suspended particles can also act as potential carriers of pathogens associated with ADD (Beaudeau 2014). Additionally, the first flush phenomenon, characterized by the mobilization of pollutants accumulated during the dry season with the onset of rainfall, may result in initial contamination peaks (Al Mamoon et al. 2019), which may be associated with temporarily increased microbial loads in water sources and increased exposure conditions, especially in hybrid systems and those using disinfection without filtration.

The low frequency of chlorination in these systems can compromise the adequate maintenance of free residual chlorine, especially during periods of heavy rainfall, when turbidity and microbial load increase. Kumpel and Nelson (2016) note that following initial contamination peaks, the system stabilizes and disinfection practices are reestablished.

The study's findings indicate that seasonality is associated with changes in contamination patterns and the occurrence of waterborne diseases, particularly in hybrid systems with operational limitations. According to theWHO (2022a), climatic events such as heavy rains can affect water quality and are associated with increased waterborne diseases. Thus, the need to strengthen WSPs is reinforced to support system resilience under variable environmental conditions.

The higher occurrence of ADD during the rainy season observed in this study reinforces the need to adopt measures that enhance the resilience of WSS to climate variability. In this context, Howard et al. (2026) emphasize that integrated catchment management approaches can help reduce the impacts of heavy rainfall on hydrological regimes and raw water quality. These approaches include nature‐based solutions such as riparian restoration, wetland conservation, catchment reforestation, and water retention measures, which can improve source water protection and reduce the transport of contaminants into WSS.

In addition to the results presented, 785 people in the study area consume water that is neither treated nor monitored, which may increase potential exposure to pathogens. This finding is consistent with Santos et al. (2023), who point to greater vulnerability to microbiological contamination in low‐income settings, especially where distribution networks have irregular connections.

In this sense, the results also highlight inequalities in access to safe water, reinforcing that exposure to risks does not occur uniformly across population groups. This discussion falls within the context of environmental justice, encompassing aspects such as access to affordable rates and high‐quality treated water. The full realization and universal fulfillment of the human right to water depend on democratic and participatory governance, as emphasized by Piccoli et al. (2016).

This study has limitations, including potential underreporting of ADD cases. Although the analysis considered only data from 2023, the authors had access to historical data from the municipal health system. This year was selected because it showed greater consistency in reporting procedures and higher data integrity, whereas platform changes in previous years compromised data comparability. This limitation highlights the challenges inherent in the use of secondary data in public health, especially regarding the standardization and reliability of information over time.

As noted by DeFelice et al. (2016), ecological studies have limitations inherent to this type of research, particularly regarding causal inference. Operational changes in the WSS may not have been fully captured, which could influence the results.

Although the observed associations were statistically significant, they should be interpreted with caution, as confounding factors may have influenced the results. Accordingly, the findings should be interpreted as evidence of associations rather than causal relationships.

Nevertheless, the study presents relevant strengths, such as the integration of spatial and epidemiological data—an approach still rarely applied in low‐ and middle‐income countries—as well as the analysis of risks associated with different WSS configurations under real‐world conditions. These aspects contribute to advancing knowledge on risks in heterogeneous water supply systems in low‐ and middle‐income countries, supporting the planning of actions in the context of climate change.

## 5 Conclusion

The results indicate that seasonality was associated with differences in ADD occurrence in Mariana, MG, with a 27% lower ADD rate during the dry season compared to the rainy season. The highest rates were observed in systems that distribute a mixture of treated and raw water, while conventional systems had the lowest rates in both periods. These findings suggest that, although more efficient in microbiological control, conventional systems are also influenced by seasonal variability.

In the context of climate variability, there is a need for standardization of operational procedures and improvements in WSS infrastructure, which may contribute to reducing adverse health outcomes under conditions of environmental stress and system vulnerability. These actions are relevant both for systems that distribute a mixture of raw and treated water, which show higher rates, and for conventional WSS, which exhibit operational limitations.

The ADD rate was 21% higher in systems that distribute a mixture of treated and raw water compared to conventional WSS, suggesting greater structural and operational vulnerability under the conditions observed in this study. In contrast, conventional systems, based on full‐cycle treatment, demonstrated greater capacity for microbiological control under routine operational conditions.

Water quality at consumption points showed no statistically significant association with the ADD rate among the served population. However, monitoring these parameters proved important for supporting corrective decision‐making, contributing to interventions that can enhance water safety during periods of heavy rainfall.

Thus, the results indicate that changes in rainfall patterns, potentially associated with climate change, should be considered a challenge to ensuring the resilience of water supply systems, regardless of structural or operational conditions. Consequently, actions such as continuous water quality monitoring, standardization of operational procedures, and control of interventions in distribution networks are important measures to support the reduction of adverse outcomes during periods of heavy rainfall in systems with structural and operational vulnerabilities.

## Author Contributions

**Isabel Francisco de Araújo Reis:** conceptualization, investigation, writing – original draft, methodology, validation, visualization, writing – review and editing, software, formal analysis, project administration, data curation, supervision. **Paula Cristine Silva Gomes:** methodology. **Thaís Barreto Santana:** methodology. **Lívia Cristina Pinto Dias:** methodology. **Marcelo de Souza Lauretto:** methodology, validation, writing – review and editing. **Maria Tereza Pepe Razzolini:** methodology, supervision, validation, writing – review and editing. **Aníbal da Fonseca Santiago:** conceptualization, methodology, validation, formal analysis, project administration, supervision, resources, writing – review and editing.

## Funding

This work was supported by Coordenação de Aperfeiçoamento de Pessoal de Nível Superior (001) and Conselho Nacional de Desenvolvimento Científico e Tecnológico (PQ) (Proc. 309529/2025‐5).

## Conflicts of Interest

The authors declare no conflicts of interest.

## Supporting information


**Table S1:1** Raw dataset used for the comparison and selection of the candidate generalized linear models (Gaussian with normal distribution and identity link function, and negative binomial with log link function).

**Table S2:** 1. Comparison of Poisson and negative binomial regression models using AIC, BIC, and deviance.
**Table S2:** 2. Results of the negative binomial regression model for acute diarrheal disease (ADD) rates. Coefficients, standard errors, z statistics, p‐values, rate ratios (RR), and 95% confidence intervals are presented.
**Figure S2:** 3. Diagnostic evaluation of the negative binomial regression model.
**Table S2:** 4. Diagnostic assessment of the negative binomial regression model.

**Table S3:** 1. Results of the generalized linear model (GLM) with a Gaussian (normal) distribution and an identity link function, using the log‐transformed ADD rate per 100,000 inhabitants as the dependent variable. The model evaluated the iAF, dry season (reference: rainy season), hybrid water supply system, and disinfection system (reference: conventional water supply system).
**Table S3:** 2. Results of the generalized linear model (GLM) with a Gaussian (normal) distribution and an identity link function, using the log‐transformed ADD rate per 100,000 inhabitants as the dependent variable. The independent variables included the hybrid water supply system and disinfection system, with the conventional water supply system as the reference category.
**Table S3:** 3. Results of the generalized linear model (GLM) with a Gaussian (normal) distribution and an identity link function, using the log‐transformed ADD rate per 100,000 inhabitants as the dependent variable. The independent variable included the disinfection water supply system, with the conventional water supply system as the reference category.
**Table S3:** 4. Results of the generalized linear model (GLM) with a Gaussian (normal) distribution and an identity link function, using the log‐transformed ADD rate per 100,000 inhabitants as the dependent variable. The independent variable included the hybrid water supply system, with the conventional water supply system as the reference category.
**Table S3:** 5. Results of the generalized linear model (GLM) with a Gaussian (normal) distribution and an identity link function, using the log‐transformed ADD rate per 100,000 inhabitants as the dependent variable. The independent variable included iAF.
**Table S3:** 6. Results of the generalized linear model (GLM) with a Gaussian (normal) distribution and an identity link function, using the log‐transformed ADD rate per 100,000 inhabitants as the dependent variable. The final model included the dry season (reference: rainy season) and the hybrid water supply system (reference: conventional water supply system), both of which were statistically significant.
**Table S3:** 7. Comparison of the AIC, BIC, deviance, and coefficient of determination (R2) for the models presented in Tables S3.1–S3.6, supporting the selection of the model with the best fit.
**Figure S3:** 1. Diagnostic evaluation of the Gaussian (normal) generalized linear model with an identity link function.

## Data Availability

The data that support the findings of this study are available from the corresponding author upon reasonable request.

## References

[wer70563-bib-0001] Ahmed, W. , R. Neller , and M. Katouli . 2005. “Evidence of Septic System Failure Determined by a Bacterial Biochemical Fingerprinting Method.” Journal of Applied Microbiology 98, no. 4: 910–920. 10.1111/j.1365-2672.2004.02522.x. 15752338

[wer70563-bib-0002] Al Mamoon, A. A. , S. Jahan , X. He , N. E. Joergensen , and A. Rahman . 2019. “First Flush Analysis Using a Rainfall Simulator on a Micro Catchment in an Arid Climate.” Science of the Total Environment 693: 133552. 10.1016/j.scitotenv.2019.07.358. 31377371

[wer70563-bib-0003] Ashbolt, N. J. 2015. “Microbial Contamination of Drinking Water and Human Health From Community Water Systems.” Current Environmental Health Reports 2, no. 1: 95–106. 10.1007/s40572-014-0037-5. 25821716 PMC4372141

[wer70563-bib-0004] Barragán, B. L. G. , M. S. Lauretto , M. T. P. Razzolini , A. C. Nardocci , and K. V. M. Sibaja . 2022. “Assessment Microbial Risks for the Presence of *Cryptosporidium* spp. and *Giardia* spp. Based on the Surveillance of the Water Supply Systems in Colombia, 2014–2018.” Water Environment Research 94: e10776. 10.1002/wer.10776. 35978464

[wer70563-bib-0005] Bataiero, M. O. , R. S. Araújo , A. C. Nardocci , et al. 2019. “Quantification of Giardia and Cryptosporidium in Surface Water: A Risk Assessment and Molecular Characterization.” Water Science and Technology: Water Supply 19, no. 6: 1823–1830. 10.2166/ws.2019.059.

[wer70563-bib-0053] Beaudeau, P. , J. Schwartz , and R. Levin . 2014. “Drinking Water Quality and Hospital Admissions of Elderly People for Gastrointestinal Illness in Eastern Massachusetts, 1998–2008.” Water Research 52: 188–198. 10.1016/j.watres.2014.01.005. 24486855

[wer70563-bib-0007] Betancourt, W. Q. , and J. B. Rose . 2004. “Drinking Water Treatment Processes for Removal of *Cryptosporidium* and *Giardia* .” Veterinary Parasitology 126: 219–234. 10.1016/j.vetpar.2004.09.002. 15567586

[wer70563-bib-0008] Brasil. Ministério da Saúde . 2021. Portaria GM/MS n° 888, de 4 de Maio de 2021: Altera o Anexo XX da Portaria de Consolidação n° 5/GM/MS, de 28 de Setembro de 2017, Para Dispor Sobre os Procedimentos de Controle e de Vigilância da Qualidade da Água Para Consumo Humano e seu Padrão de Potabilidade. Diário Oficial da União. https://saec.sp.gov.br/wp‐content/uploads/2025/09/Portaria‐GM‐MS‐888‐DE‐04‐DE‐MAIO‐DE‐2021.pdf.

[wer70563-bib-0009] Brasil. Ministério da Saúde. Fundação Nacional de Saúde . 2019. Programa Nacional de Saneamento Rural Fundação Nacional de Saúde. https://www.gov.br/funasa/pt‐br/centrais‐de‐conteudo/publicacoes/engenharia‐de‐saude‐publica/programa‐nacional‐de‐saneamento‐rural.

[wer70563-bib-0010] Button, K. S. , J. P. A. Ioannidis , C. Mokrysz , et al. 2013. “Power Failure: Why Small Sample Size Undermines the Reliability of Neuroscience.” Nature Reviews Neuroscience 14, no. 5: 365–376. 10.1038/nrn3475. 23571845

[wer70563-bib-0011] Carlton, E. J. , A. P. Woster , P. DeWitt , R. S. Goldstein , and K. Levy . 2016. “A Systematic Review and Meta‐Analysis of Ambient Temperature and Diarrhoeal Diseases.” International Journal of Epidemiology 45, no. 1: 117–130. 10.1093/ije/dyv296. 26567313 PMC4881833

[wer70563-bib-0057] Centro Nacional de Monitoramento e Alertas de Desastres Naturais . 2025. Boletim Pluviométrico – Estação Mariana/MG: Março de 2023. CEMADEN. https://mapainterativo.cemaden.gov.br/.

[wer70563-bib-0013] Curriero, F. C. , J. A. Patz , J. B. Rose , and S. Lele . 2001. “The Association Between Extreme Precipitation and Waterborne Disease Outbreaks in the United States, 1948–1994.” American Journal of Public Health 91, no. 8: 1194–1199. 10.2105/AJPH.91.8.1194. 11499103 PMC1446745

[wer70563-bib-0014] de Castro, R. S. , V. R. N. Cruvinel , and J. L. M. da Oliveira . 2019. “Correlação Entre Qualidade da Água e Ocorrência de Diarreia e Hepatite A no Distrito Federal/Brasil.” Saúde em Debate 43, no. spe3: 8–19. 10.1590/0103-11042019S301.

[wer70563-bib-0015] DeFelice, N. B. , J. E. Johnston , and J. M. Gibson . 2016. “Reducing Emergency Department Visits for Acute Gastrointestinal Illnesses in North Carolina (USA) by Extending Community Water Service.” Environmental Health Perspectives 124: 1583–1591. 10.1289/EHP160. 27203131 PMC5047767

[wer70563-bib-0016] Egorov, A. , T. Ford , A. Tereschenko , N. Drizhd , I. Segedevich , and V. Fourman . 2002. “Deterioration of Drinking Water Quality in the Distribution System and Gastrointestinal Morbidity in a Russian City.” International Journal of Environmental Health Research 12, no. 3: 221–233. 10.1080/09603/202/000000989. 12396523

[wer70563-bib-0056] Ercumen, A. , J. S. Gruber , and J. M. Colford, Jr . 2014. “Water Distribution System Deficiencies and Gastrointestinal Illness: A Systematic Review and Meta‐Analysis.” Environmental Health Perspectives 122, no. 7: 651–660. 10.1289/ehp.1306912. 24659576 PMC4080524

[wer70563-bib-0018] Ferreira, D. C. , I. Graziele , R. C. Marques , and J. Gonçalves . 2021. “Investment in Drinking Water and Sanitation Infrastructure and Its Impact on Waterborne Diseases Dissemination: The Brazilian Case.” Science of the Total Environment 779: 146279. 10.1016/j.scitotenv.2021.146279. 33743461

[wer70563-bib-0019] Focus, A. L. , A. Nazir , and O. Uwishema . 2023. “The Devastating Effect of Cyclone Freddy Amidst the Deadliest Cholera Outbreak in Malawi: A Double Burden for an Already Weak Healthcare System—Short Communication.” Annals of Medicine and Surgery 85, no. 7: 3761–3763. 10.1097/MS9.0000000000000961. 37427161 PMC10328608

[wer70563-bib-0020] Fonseca, P. A. M. , S. S. Hacon , V. L. Reis , D. Costa , and I. F. Brown . 2016. “Using Satellite Data to Study the Relationship Between Rainfall and Diarrheal Diseases in a Southwestern Amazon Basin.” Ciência & Saúde Coletiva 21: 731–742. 10.1590/1413-81232015213.20162015. 26960086

[wer70563-bib-0021] GBD 2021 Diarrhoeal Diseases Collaborators . 2025. “Global, Regional, and National Age‐Sex‐Specific Burden of Diarrhoeal Diseases, Their Risk Factors, and Aetiologies, 1990–2021, for 204 Countries and Territories: A Systematic Analysis for the Global Burden of Disease Study 2021.” Lancet Infectious Diseases 25, no. 5: 519–536. 10.1016/S1473-3099(24)00691-1. 39708822 PMC12018300

[wer70563-bib-0023] Howard, G. , L. Beevers , K. Charles , and A. Nijhawan . 2026. “The Vulnerability and Resilience of Drinking Water Systems to Extreme Weather Events and Future Climate Change.” Current Environmental Health Reports 13: 5. 10.1007/s40572-026-00524-y. 41642556 PMC12876451

[wer70563-bib-0024] Institute for Health Metrics and Evaluation . 2021. Global Burden of Disease Study 2021 (GBD 2021): Incidence of Diarrheal Diseases https://ourworldindata.org/grapher/incidence‐of‐diarrheal‐diseases.

[wer70563-bib-0055] Instituto Brasileiro de Geografia e Estatística . 2023. Censo Demográfico 2022: População e Domicílios: Primeiros Resultados do Universo. IBGE. https://sidra.ibge.gov.br/pesquisa/censo‐demografico/demografico‐2022/primeiros‐resultados‐populacao‐e‐domicilios.

[wer70563-bib-0026] Kumpel, E. , and K. L. Nelson . 2016. “Intermittent Water Supply: Prevalence, Practice, and Microbial Water Quality.” Environmental Science & Technology 50, no. 2: 542–553. 10.1021/acs.est.5b03973. 26670120

[wer70563-bib-0027] LeChevallier, M. W. , R. Gullick , M. Karim , M. Friedman , and J. Funk . 2003. “The Potential for Health Risks From Intrusion of Contaminants Into the Distribution System From Pressure Transients.” Journal of Water and Health 1: 3–14. 10.2166/wh.2003.0002. 15384268

[wer70563-bib-0028] Levy, K. , A. P. Woster , R. S. Goldstein , and E. J. Carlton . 2016. “Untangling the Impacts of Climate Change on Waterborne Diseases: A Systematic Review of Relationships Between Diarrheal Diseases and Temperature, Rainfall, Flooding, and Drought.” Environmental Science & Technology 50, no. 10: 4905–4922. 10.1021/acs.est.5b06186. 27058059 PMC5468171

[wer70563-bib-0054] Mazurek, L. , C. B. Miguel , H. P. Pinto Neto , et al. 2025. “Impact of Transitioning to Treated Water on Diarrhea Reduction: A Cross‐Sectional and Ecological Study in Southwestern Goiás, Brazil.” International Journal of Environmental Research and Public Health 22: 436. 10.3390/ijerph22030436. 40238514 PMC11942217

[wer70563-bib-0030] Meisen, M. N. , N. Bohn , L. B. B. Tavares , and A. Pinheiro . 2011. “Análise de Correlação da Ocorrência de Doenças Diarreicas Agudas (DDA) com a Qualidade da Água Para Consumo Humano no Município de Pouso Redondo–SC.” Revista de Estudos Ambientais 13, no. 2: 57–67. https://ojsrevista.furb.br/ojs/index.php/rea/article/view/2673/1803.

[wer70563-bib-0031] Orner, K. D. , A. Calvo , J. Zhang , and J. R. Mihelcic . 2017. “Effectiveness of In‐Line Chlorination in a Developing World Gravity‐Flow Water Supply.” Waterlines 36, no. 2: 167–182. 10.3362/1756-3488.16-00016.

[wer70563-bib-0032] Piccoli, A. S. , D. C. Kligerman , S. C. Cohen , and R. F. Assumpção . 2016. “A Educação Ambiental Como Estratégia de Mobilização Social Para o Enfrentamento da Escassez de Água.” Ciência & Saúde Coletiva 21: 797–808. 10.1590/1413-81232015213.26852015. 26960092

[wer70563-bib-0034] Prüss‐Ustün, A. , J. Bartram , T. Clasen , et al. 2014. “Burden of Disease From Inadequate Water, Sanitation and Hygiene in Low‐ and Middle‐Income Settings: A Retrospective Analysis of Data From 145 Countries.” Tropical Medicine & International Health 19, no. 8: 894–905. 10.1111/tmi.12329. 24779548 PMC4255749

[wer70563-bib-0035] Queiroz, J. T. M. , L. Heller , and S. R. Silva . 2009. “Análise da Correlação de Ocorrência da Doença Diarreica Aguda com a Qualidade da Água Para Consumo Humano no Município de Vitória‐ES.” Saúde e Sociedade 18, no. 3: 479–489. 10.1590/S0104-12902009000300012.

[wer70563-bib-0037] Santos, N. G. N. , L. C. Silva , G. H. M. Guidone , et al. 2023. “Water Quality Monitoring in Southern Brazil and the Assessment of Risk Factors Related to Contamination by Coliforms and *Escherichia coli* .” Journal of Water and Health 21, no. 10: 1550–1561. 10.2166/wh.2023.182. 37902208

[wer70563-bib-0038] Santos, R. H. C. , D. L. Barbosa , and A. C. L. Rodrigues . 2021. “Análise dos Modos e Efeitos de Falhas no Sistema de Abastecimento de Água do Município de Bananeiras‐PB: Uma Abordagem da Captação à Rede de Distribuição.” Revista Ibero‐Americana de Ciências Ambientais 12, no. 8: 469–484. https://www.researchgate.net/publication/359740980_Analise_dos_modos_e_efeitos_de_falhas_no_sistema_de_abastecimento_de_agua_do_municipio_de_Bananeiras‐PB_uma_abordagem_da_captacao_a_rede_de_distribuicao.

[wer70563-bib-0039] Sato, M. I. Z. , A. T. Galvani , J. A. Padula , et al. 2013. “Assessing the Infection Risk of Giardia and Cryptosporidium in Public Drinking Water Delivered by Surface Water Systems in São Paulo State, Brazil.” Science of the Total Environment 442: 389–396. 10.1016/j.scitotenv.2012.09.077. 23178841

[wer70563-bib-0040] Secretaria Municipal de Saúde de Mariana . 2023. Sistema de Monitoramento de Doenças Diarreicas Agudas do Município de Mariana, Minas Gerais [Base de Dados]. Prefeitura Municipal de Mariana.

[wer70563-bib-0041] Serviço Autônomo de Água e Esgoto . 2025. Relatório Anual de Qualidade da Água https://saaemariana.mg.gov.br/relatorio‐anual‐qualidade‐da‐agua/.

[wer70563-bib-0042] Souza, L. A. 2004. Diagnóstico do Meio Físico Como Contribuição ao Ordenamento Territorial do Município de Mariana (MG) [Dissertação de mestrado. Universidade Federal de Ouro Preto Repositório Institucional da UFOP. https://www.repositorio.ufop.br/handle/123456789/6436.

[wer70563-bib-0043] Su, B. , W. Dong , T. Jiang , and Z. W. Kundzewicz . 2025. “Further Efforts in Climate Change Adaptation and Mitigation Are Indispensable.” Innovation 6, no. 8: 100888. 10.1016/j.xinn.2025.100888. 40814352 PMC12347099

[wer70563-bib-0044] Tinker, S. C. , C. L. Moe , M. Klein , et al. 2009. “Drinking Water Residence Time in Distribution Networks and Emergency Department Visits for Gastrointestinal Illness in Metro Atlanta, Georgia.” Journal of Water and Health 7, no. 2: 332–343. 10.2166/wh.2009.022. 19240359 PMC3743167

[wer70563-bib-0045] United Nations Children's Fund , and World Health Organization . 2023. “Progress on Household Drinking‐Water, Sanitation and Hygiene 2000–2022: Special Focus on Gender.” https://www.who.int/publications/i/item/9789240076921.

[wer70563-bib-0046] Uwishema, O. , D. S. Masunga , K. M. Naisikye , et al. 2023. “Impacts of Environmental and Climatic Changes on Future Infectious Diseases.” International Journal of Surgery 109, no. 2: 167–170. 10.1097/JS9.0000000000000160. 36799840 PMC10389506

[wer70563-bib-0047] Vieira, J. M. P. 2018. Água e Saúde Pública. Edições Sílabo.

[wer70563-bib-0048] World Health Organization . 2014. “Preventing Diarrhoea Through Better Water, Sanitation and Hygiene: Exposures and Impacts in Low‐ and Middle‐Income Countries.” https://www.who.int/publications/i/item/9789241564823.

[wer70563-bib-0049] World Health Organization 2022a. “Addenda to the WHO WASH Strategy 2018–2023: WASH and Climate Change Adaptation and Mitigation for Health, 2023–2030.” https://www.who.int/publications/m/item/wash‐and‐climate‐change‐adaptation‐and‐mitigation‐for‐health‐‐2023‐2030.

[wer70563-bib-0050] World Health Organization . 2022b. “Guidelines for Drinking‐Water Quality: Fourth Edition Incorporating the First and Second Addenda.” https://www.who.int/publications/i/item/9789240045064. 35417116

[wer70563-bib-0051] World Health Organization . 2024. “The Top 10 Causes of Death.” https://www.who.int/news‐room/fact‐sheets/detail/the‐top‐10‐causes‐of‐death.

