# Supplementary material for: Acute Diarrheal Disease Associated With Climate Variability and Water Supply System Configuration: An Ecological Study in Brazil
Source: Water Environ Res. 2026 Sep 9;98(9):e70563. doi: 10.1002/wer.70563 (PMC13557016; doi:10.1002/wer.70563)
Supplement: Supplementary file 1 [file WER-98-e70563-s001.docx]

**Supplementary Material S3**

**Gaussian generalized linear model: model specification, model selection, and diagnostic evaluation**

This supplementary material presents the results of all generalized linear models with a Gaussian (normal) distribution and an identity link function evaluated during the model selection process, including models that were not retained in the final model because they did not improve model fit or were not statistically significant. It also presents the diagnostic evaluation of the Gaussian generalized linear model with an identity link function, which was selected as the final model based on the model selection procedure.

A generalized linear model (GLM) with a Gaussian (normal) distribution and identity link function was fitted. Because the objective of the study was to compare the occurrence of ADD among water supply systems serving different population sizes, the response variable was redefined as the base-10 logarithm of the ADD incidence rate per 100,000 inhabitants (LogT).

Model adequacy was assessed through graphical inspection of Pearson residuals versus fitted values, normal Q–Q plots of Pearson residuals, deviance residuals versus fitted values, normal Q–Q plots of deviance residuals, standardized residuals versus fitted values, normal Q–Q plots of standardized residuals, scale–location plots, and residuals versus leverage plots, using diagnostic functions implemented in R (R Core Team, 2022). As these diagnostic procedures indicated that the model assumptions were adequately satisfied, the Gaussian GLM was adopted as the final model for the study.

The generalized linear model (GLM) with a Gaussian (normal) distribution and identity link function is presented in Equation S3.1.

$log\left( {10}^{5} \right)=\beta_{0}+\beta_{1}T_{H}+\beta_{2}T_{DS}+\beta_{3}E_{c}+\beta_{4}C_{iaf}+\varepsilon_{i}$	(S3.1)

Where:
$loglog \left( {10}^{5} \right)$: base-10 logarithm of the ADD case rate per 100,000 inhabitants in group i (dependent variable)

β_0_ : model intercept;
$\beta_{1},\beta_{2},\beta_{3},\beta_{4}$: coefficients associated with the independent variables;
$T_{H}$: indicator variable for hybrid systems;
$T_{DS}$: indicator variable for simple disinfection systems;
$E_{c}$: indicator variable for the dry season;
$C_{iaf}$: frequency of non-compliant analyses in group *i*;
$\varepsilon_{i}$: random error term, assumed to follow a normal distribution with mean zero and constant variance.

The conventional water supply system was used as the reference category for water supply system type, and the rainy season was used as the reference category for season.

The model was fitted using R version 4.2 (R Core Team, 2022) within the RStudio environment (Posit, 2024).

**Model specification and selection procedure**

Initially, a full model was specified including all explanatory variables described in Equation S3.1. These variables comprised the frequency of non-compliant analyses (iAF), the dry season (with the rainy season as the reference category), the hybrid water supply system, and the disinfection-only water supply system (with the conventional water supply system as the reference category).

Subsequently, alternative model specifications were evaluated by fitting different combinations of explanatory variables to assess their individual and joint contributions. The following specifications were considered: (i) the full model (iAF, dry season, hybrid water supply system, and disinfection-only water supply system); (ii) a model including only the water supply system variables (hybrid and disinfection-only systems); (iii) a model including only the disinfection-only water supply system; (iv) a model including only the hybrid water supply system; (v) a model including only the iAF; and (vi) a model including the hybrid water supply system and the dry season.

The results of all candidate models evaluated during the model selection process are presented in Tables S3.1–S3.6. The final model included the hybrid water supply system and dry season variables because they were statistically significant and provided the best overall model fit. Model selection was based on comparisons of the Akaike Information Criterion (AIC), Bayesian Information Criterion (BIC), deviance, and the coefficient of determination (R²) across models (i–vi), with the aim of identifying and justifying the model that achieved the best overall performance.

Statistical significance was defined as *p* < 0.05. The explanatory variables were selected based on the study hypothesis and epidemiological plausibility; therefore, no automated variable selection procedure was applied. The statistical significance of the model coefficients was assessed using the Wald test, and variables with *p* < 0.05 in the final model were considered statistically significant.

No temporal lag was applied between the water quality indicators and ADD cases because the analysis was conducted using data aggregated by water supply system and seasonal period. The objective was to evaluate ecological associations between the characteristics of the water supply systems, water quality indicators, and the occurrence of ADD.

Because the response variable was modeled using the base-10 logarithm of the ADD rate, regression coefficients (β) were back-transformed using ${10}^{\beta}$to facilitate interpretation on the original scale. The back-transformed coefficients (${10}^{\beta}$) represent multiplicative changes in the ADD rate associated with each explanatory variable relative to the reference category. Percentage changes were calculated as $\left( {10}^{\beta}-1 \right)\times100$, with positive values indicating higher ADD rates and negative values indicating lower ADD rates.

Because the response variable was calculated from monthly data aggregated by water supply system, no ADD occurrence rates equal to zero were observed. Therefore, the base-10 logarithmic transformation could be applied directly to the response variable without the need to add a constant before transformation.

**Results of the generalized linear models**

Table S3.1. Results of the generalized linear model (GLM) with a Gaussian (normal) distribution and an identity link function, using the log-transformed ADD rate per 100,000 inhabitants as the dependent variable. The model evaluated the iAF, dry season (reference: rainy season), hybrid water supply system, and disinfection system (reference: conventional water supply system).

| Variable | Coefficient β | Standard Error | t value | p value | exp(β) | 95% CI Lower | 95% CI Upper |
| --- | --- | --- | --- | --- | --- | --- | --- |
| (Intercept) | 3.6400 | 0.1000 | 35.1300 | 0.0000 | 38.1900 | 31.1700 | 46.8000 |
| Hybrid | 0.1600 | 0.1200 | 1.3600 | 0.1840 | 1.1700 | 0.9300 | 1.4700 |
| Disinfection | -0.0200 | 0.1200 | -0.2000 | 0.8440 | 0.9700 | 0.7600 | 1.2500 |
| Dry season | -0.3000 | 0.0800 | -3.5900 | 0.0010 | 0.7400 | 0.6200 | 0.8700 |
| *IAF* | -0.0006 | 0.0010 | -0.6500 | 0.5190 | 0.9900 | 0.9900 | 1.0000 |

Note: exp(β) = exponentiated coefficients; CI = confidence interval.

Table S3.2. Results of the generalized linear model (GLM) with a Gaussian (normal) distribution and an identity link function, using the log-transformed ADD rate per 100,000 inhabitants as the dependent variable. The independent variables included the hybrid water supply system and disinfection system, with the conventional water supply system as the reference category.

| Variable | Coefficient β | Standard Error | t value | p value | exp(β) | 95% CI Lower | 95% CI Upper |
| --- | --- | --- | --- | --- | --- | --- | --- |
| (Intercept) | 3.4900 | 0.1100 | 30.9100 | 0.0000 | 32.7900 | 26.2800 | 40.9000 |
| Hybrid | 0.1800 | 0.1400 | 1.2900 | 0.2100 | 1.2000 | 0.9100 | 1.5700 |
| Disinfection | -0.0565 | 0.1400 | -0.4100 | 0.6900 | 0.9400 | 0.7200 | 1.2400 |

Note: exp(β) = exponentiated coefficients; CI = confidence interval.

Table S3.3. Results of the generalized linear model (GLM) with a Gaussian (normal) distribution and an identity link function, using the log-transformed ADD rate per 100,000 inhabitants as the dependent variable. The independent variable included the disinfection water supply system, with the conventional water supply system as the reference category.

| Variable | Coefficient β | Standard Error | t value | p value | exp(β) | 95% CI Lower | 95% CI Upper |
| --- | --- | --- | --- | --- | --- | --- | --- |
| (Intercept) | 3.610 | 0.070 | 54.720 | 0.000 | 36.940 | 32.460 | 42.040 |
| Disinfection | -0.170 | 0.100 | -1.680 | 0.103 | 0.840 | 0.680 | 1.030 |

Note: exp(β) = exponentiated coefficients; CI = confidence interval.

Table S3.4. Results of the generalized linear model (GLM) with a Gaussian (normal) distribution and an identity link function, using the log-transformed ADD rate per 100,000 inhabitants as the dependent variable. The independent variable included the hybrid water supply system, with the conventional water supply system as the reference category.

| Variable | Coefficient β | Standard Error | t value | p value | exp(β) | 95% CI Lower | 95% CI Upper |
| --- | --- | --- | --- | --- | --- | --- | --- |
| (Intercept) | 3.45 | 0.06 | 53.77 | 0.00 | 31.57 | 27.84 | 35.81 |
| Hybrid | 0.22 | 0.10 | 2.13 | 0.04 | 1.24 | 1.02 | 1.51 |

Note: exp(β) = exponentiated coefficients; CI = confidence interval.

Table S3.5. Results of the generalized linear model (GLM) with a Gaussian (normal) distribution and an identity link function, using the log-transformed ADD rate per 100,000 inhabitants as the dependent variable. The independent variable included iAF.

| Variable | Coefficient β | Standard Error | t value | p value | exp(β) | 95% CI Lower | 95% CI Upper |
| --- | --- | --- | --- | --- | --- | --- | --- |
| (Intercept) | 3.5700 | 0.0580 | 61.6600 | 0.0000 | 35.4900 | 31.6800 | 39.7500 |
| *IAF* | -0.0013 | 0.0011 | -1.2200 | 0.2340 | 0.9900 | 0.9900 | 1.0000 |

Note: exp(β) = exponentiated coefficients; CI = confidence interval.

Table S3.6**.** Results of the generalized linear model (GLM) with a Gaussian (normal) distribution and an identity link function, using the log-transformed ADD rate per 100,000 inhabitants as the dependent variable. The final model included the dry season (reference: rainy season) and the hybrid water supply system (reference: conventional water supply system), both of which were statistically significant.

| Variable | Coefficient (β) | Standard Error | t value | p value | exp(β) | 95% CI Lower | 95% CI Upper |
| --- | --- | --- | --- | --- | --- | --- | --- |
| Intercept | 3.610 | 0.070 | 53.450 | < 0.001 | 36.820 | 32.260 | 42.020 |
| Hybrid | 0.190 | 0.080 | 2.260 | 0.032 | 1.210 | 1.030 | 1.430 |
| Dry season | −0.310 | 0.080 | −3.710 | 0.001 | 0.730 | 0.620 | 0.860 |

Note: exp(β) = exponentiated coefficients; CI = confidence interval.

Table S3.7. Comparison of the AIC, BIC, deviance, and coefficient of determination (R²) for the models presented in Tables S3.1–S3.6, supporting the selection of the model with the best fit.

| Model | AIC | BIC | Deviance | R² |
| --- | --- | --- | --- | --- |
| Table S3.1 | 3.90 | 12.31 | 1.34 | 0.445 |
| Table S3.2 | 12.85 | 18.45 | 2.06 | 0.145 |
| Table S3.3 | 12.65 | 16.86 | 2.19 | 0.092 |
| Table S3.4 | 11.03 | 15.24 | 2.08 | 0.140 |
| Table S3.5 | 14.01 | 18.21 | 2.29 | 0.050 |
| Table S3.6 | 0.69 | 6.30 | 1.38 | 0.430 |

Figure S3.1**.** Diagnostic evaluation of the Gaussian (normal) generalized linear model with an identity link function.
