# Supplementary material for: Acute Diarrheal Disease Associated With Climate Variability and Water Supply System Configuration: An Ecological Study in Brazil
Source: Water Environ Res. 2026 Sep 9;98(9):e70563. doi: 10.1002/wer.70563 (PMC13557016; doi:10.1002/wer.70563)
Supplement: Supplementary file 2 [file WER-98-e70563-s002.docx]

**Supplementary Material S1**

Table S1.1. Raw dataset used for the comparison and selection of the candidate generalized linear models (Gaussian with normal distribution and identity link function, and negative binomial with log link function).

| WSS | Treatment type | Cases | Population | Rate | LogT | Season | *IAF* |
| --- | --- | --- | --- | --- | --- | --- | --- |
| scetamatadouro | Conventional | 33.00000 | 758.00000 | 4353.56201 | 3.6388447 | Rainy season | 0.00001 |
| scetamatadouro | Conventional | 15.00000 | 758.00000 | 1978.89182 | 3.2964221 | Dry season | 0.00001 |
| scetasantarita | Conventional | 248.0000 | 6281.00000 | 3948.415857 | 3.5964229 | Rainy season | 0.00001 |
| scetasantarita | Conventional | 96.00000 | 6281.00000 | 1528.419042 | 3.1842424 | Dry season | 0.00001 |
| scetasul | Conventional | 272.00000 | 4149.00000 | 6555.796577 | 3.8166255 | Rainy season | 0.00001 |
| scetasul | Conventional | 106.00000 | 4149.00000 | 2554.83249 | 3.4073624 | Dry season | 0.00001 |
| sdscartuxadulico | Disinfection | 58.00000 | 2186.00000 | 2653.247941 | 3.4237778 | Rainy season | 0.00001 |
| sdscartuxadulico | Disinfection | 25.00000 | 2186.00000 | 1143.641354 | 3.0582899 | Dry season | 198.79518 |
| sdsefigenia | Disinfection | 27.00000 | 2277.00000 | 1185.770751 | 3.0740007 | Rainy season | 93.02325 |
| sdsefigenia | Disinfection | 19.00000 | 2277.00000 | 834.4312692 | 2.9213906 | Dry season | 119.04762 |
| sdsgogo | Disinfection | 497.00000 | 9190.00000 | 5408.052231 | 3.7330409 | Rainy season | 0.00001 |
| sdsgogo | Disinfection | 235.00000 | 9190.00000 | 2557.127312 | 3.4077524 | Dry season | 10.25641 |
| sdsliberdade | Disinfection | 6.00000 | 195.00000 | 3076.923077 | 3.4881166 | Rainy season | 0.00001 |
| sdsliberdade | Disinfection | 5.00000 | 195.00000 | 2564.102564 | 3.4089354 | Dry season | 0.00001 |
| sdsmaqinconf | Disinfection | 96.00000 | 1959.00000 | 4900.459418 | 3.6902368 | Rainy season | 0.00001 |
| sdsmaqinconf | Disinfection | 25.00000 | 1959.00000 | 1276.161307 | 3.1059056 | Dry season | 0.00001 |
| sdspantera | Disinfection | 138.00000 | 997.00000 | 13841.52457 | 4.1411839 | Rainy season | 143.000000 |
| sdspantera | Disinfection | 56.00000 | 997.00000 | 5616.850552 | 3.7494929 | Dry season | 27.77777 |
| shcristalmaquiné | Hybrid | 649.00000 | 7637.00000 | 8498.101349 | 3.9293219 | Rainy season | 40.17857 |
| shcristalmaquiné | Hybrid | 320.00000 | 7637.00000 | 4190.127013 | 3.6222272 | Dry season | 5.37634 |
| shdelrey | Hybrid | 115.00000 | 1367.00000 | 8412.582297 | 3.9249293 | Rainy season | 30.30303 |
| shdelrey | Hybrid | 43.00000 | 1367,00000 | 3145,57425 | 3,4976999 | Dry season | 16.12903 |
| shetamatadouro | Hybrid | 55.00000 | 785,00000 | 7006,369427 | 3,845493 | Rainy season | 0.00001 |
| shetamatadouro | Hybrid | 35.00000 | 785,00000 | 4458,598726 | 3,6491984 | Dry season | 0.00001 |
| shetasantarita | Hybrid | 234.00000 | 4684,00000 | 4995,730145 | 3,698599 | Rainy season | 0.00001 |
| shetasantarita | Hybrid | 124.00000 | 4684,00000 | 2647,309991 | 3,4228048 | Dry season | 0.00001 |
| shetaseminario | Hybrid | 119.00000 | 2044.00000 | 5821.917808 | 3,7650661 | Rainy season | 0.00001 |
| shetaseminario | Hybrid | 70.00000 | 2044.00000 | 3424.657534 | 3,5346171 | Dry season | 0.00001 |
| shetasul | Hybrid | 230.00000 | 3910.00000 | 5882.352941 | 3,7695511 | Rainy season | 9.00900 |
| shetasul | Hybrid | 91.00000 | 3910.00000 | 2327.365729 | 3,3668646 | Dry season | 0.00001 |
