# Supplementary material for: Acute Diarrheal Disease Associated With Climate Variability and Water Supply System Configuration: An Ecological Study in Brazil
Source: Water Environ Res. 2026 Sep 9;98(9):e70563. doi: 10.1002/wer.70563 (PMC13557016; doi:10.1002/wer.70563)
Supplement: Supplementary file 3 [file WER-98-e70563-s003.docx]

**Supplementary Material S2**

**Selection and diagnostic evaluation of count regression models**

This supplementary material presents the selection and diagnostic evaluation of count regression models used to investigate the association between drinking water supply characteristics and acute diarrheal disease (ADD) cases. Poisson and negative binomial regression models were evaluated using the number of ADD cases as the response variable and the logarithm of the population served as an offset.

Initially, a Poisson regression model was fitted, as it is a standard reference model for count data (Nelder & Wedderburn, 1972; McCullagh & Nelder, 1989, as cited in Zeileis et al., 2008). However, because the variance of the response variable (number of ADD cases) exceeded its mean, indicating overdispersion, a negative binomial regression model was also fitted. The same explanatory variables considered in the study were included in the count regression models.

Negative binomial regression is recommended when overdispersion is present in count data (Nelder & Wedderburn, 1972; McCullagh & Nelder, 1989, as cited in Zeileis et al., 2008). Model performance was compared using the Akaike Information Criterion (AIC), Bayesian Information Criterion (BIC), and deviance, following the approach described by Fenta et al. (2020), to identify the model that showed the best performance for the count data.

The negative binomial model showed better performance than the Poisson model according to these goodness-of-fit criteria. Therefore, regression coefficients, rate ratios (RRs), and corresponding 95% confidence intervals were estimated for the negative binomial model.

Model adequacy was assessed using simulation-based residual diagnostics according to Hartig (2022), including the Kolmogorov–Smirnov test for residual uniformity, the outlier test, the dispersion test, and graphical inspection of simulated residuals versus fitted values.

Although the negative binomial model showed better performance than the Poisson model according to the goodness-of-fit criteria, the diagnostic evaluation indicated significant underdispersion (dispersion test, p = 0.040), suggesting that the residual variability was lower than expected under the fitted model. This finding indicated that the model did not adequately represent the underlying data structure, justifying the evaluation of an alternative modeling approach.

The comparison between the Poisson and negative binomial models is presented in Table S2.1.

Table S2.1. Comparison of Poisson and negative binomial regression models using AIC, BIC, and deviance.

| Model | AIC | BIC | “Deviance” |
| --- | --- | --- | --- |
| Poisson | 327.72 | 348.74 | 112.99 |
| Negative binomial | 281.41 | 303.83 | 28.20 |

The regression results for the negative binomial model are presented in Table S2.2.

**Table S2.2. Results of the negative binomial regression model for acute diarrheal disease (ADD) rates. Coefficients, standard errors, z statistics, p-values, rate ratios (RR), and 95% confidence intervals are presented.**

| Variable | Coefficient (β) | Standard Error | z value | p-value | RR = exp(β) | 95% CI – Lower | 95% CI – Upper |
| --- | --- | --- | --- | --- | --- | --- | --- |
| Intercept | -3.057 | 0.207 | -14.777 | <0.001 | 0.047 | 0.032 | 0.072 |
| *IAF* | 0.001 | 0.002 | 0.444 | 0.657 | 1.001 | 0.997 | 1.005 |
| Disinfection | 0.031 | 0.256 | 0.122 | 0.903 | 1.032 | 0.618 | 1.707 |
| Hybrid | 0.394 | 0.232 | 1.701 | 0.089 | 1.483 | 0.930 | 2.318 |
| Dry season | -0.783 | 0.171 | -4.586 | <0.001 | 0.457 | 0.325 | 0.642 |

The diagnostic plots are presented in Figure S2.3, and the corresponding diagnostic test results are presented in Table S2.4.

Figure S2.3. Diagnostic evaluation of the negative binomial regression model.

Table S2.4. Diagnostic assessment of the negative binomial regression model.

| Diagnostic test | p-value | Interpretation |
| --- | --- | --- |
| Kolmogorov–Smirnov test (uniformity of simulated residuals) | 0.706 | No significant deviation from the expected residual distribution |
| Outlier test | 1.000 | No significant outliers detected |
| Dispersion test | 0.040 | Significant underdispersion detected |
| Residuals versus fitted values | — | No systematic pattern observed |
